# Repeated antitachycardia pacing using the intrinsic algorithm in the ventricular fibrillation zone: Is it a risk or benefit?

**DOI:** 10.1016/j.hrcr.2024.11.007

**Published:** 2024-11-12

**Authors:** Yusuke Ikada, Masato Okada, Yuki Masuda, Koji Tanaka, Nobuaki Tanaka

**Affiliations:** Cardiovascular Center, Sakurabashi Watanabe Advanced Healthcare Hospital, Osaka, Japan

**Keywords:** Antitachycardia pacing, Implantable cardioverter-defibrillator, Intrinsic antitachycardia pacing, Ventricular fibrillation, Ventricular tachycardia

**Key Teaching Points**

• Antitachycardia pacing (ATP) is an established pain-free method for terminating ventricular tachycardia (VT). However, it may increase the risk of VT acceleration, syncope, or delayed defibrillation.
• Previously, ATP in the ventricular fibrillation (VF) zone had been limited to a maximum of 2 sequences. Currently, with the introduction of the intrinsic algorithm (intrinsic ATP), the latest implantable cardioverter-defibrillator model (Cobalt XP; Medtronic, Minneapolis, MN) can support up to 4 ATP sequences in the VF zone.
• Repeated intrinsic ATP therapy accelerated but ultimately terminated VTs in the VF zone in our 2 cases, suggesting a dual nature of the therapy with both proarrhythmic and antiarrhythmic effects.
• Large-scale multicenter studies are necessary to accurately assess the efficacy and safety of repeated intrinsic ATPs in the VF zone.

## Introduction

Minimizing shocks is an important aspect of care for patients with implantable cardioverter-defibrillators (ICDs). Antitachycardia pacing (ATP) offers an effective, shock-free method for terminating ventricular tachycardias (VTs).^1^ Several studies have revealed that ATP is more effective in terminating slower VTs with a prolonged VT cycle length (VTCL) than fast VTs with a short VTCL.^1^^,^^2^ Due to the potential risk of delayed defibrillation, programming repeated ATP therapies in the ventricular fibrillation (VF) zone remains controversial.^3^^,^^4^ Traditionally, the delivery of 2 ATP sequences (before and during charging) has been set as the maximal setting. However, with the emergence of a novel auto-programmed ATP algorithm (intrinsic ATP [iATP]), a maximum of 4 ATP sequences are available in the VF zone in the latest ICD model (Cobalt XP, Medtronic, Minneapolis, MN). Here, we present 2 cases in which repeated iATP therapy initially accelerated but ultimately terminated VTs in the VF zone.

## Case reports

### Case 1

**Figure 1:**
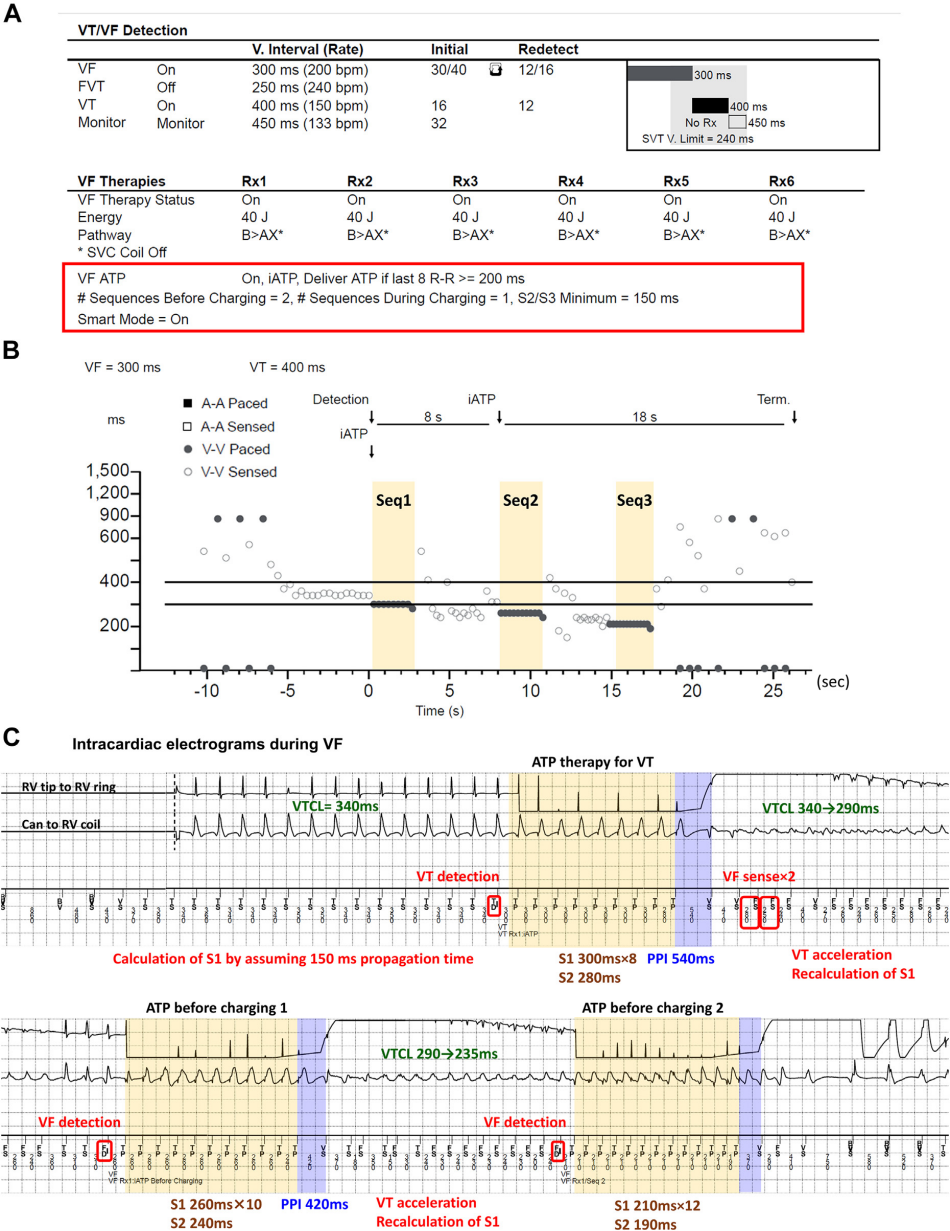
**A:** Parameter settings for tachycardia therapy in case 1. Antitachycardia pacing (ATP) was actively programmed even in the ventricular fibrillation (VF) zone (*red box line*). Three sequences of intrinsic ATP were set to be delivered if the last 8 R-R intervals were ≥200 ms. **B:** The Medtronic (Minneapolis, MN) programmer shows the successful administration of repeated intrinsic ATP (ie, ATP for stable ventricular tachycardia [VT] ×1 and ATP for fast VT in the VF zone ×2) (*yellow bands*), which eventually led to tachycardia termination and prevented the occurrence of shock. **C:** Intracardiac electrograms during the VF episode. The *yellow* and *blue bands* represent the electrograms recorded during ATP and post-pacing intervals, respectively. In this case, the initial ATP (88% of VT cycle length [VTCL] assuming 150 ms travel time) accelerated the VT, shortening the VTCL and reclassifying the arrhythmia as VF. Although the number of S1 pulses was recalculated, the second sequence did not terminate the tachycardia and further accelerated it. Ultimately, the third ATP sequence, consisting of 12 S1 pulses with a pacing cycle length of 210 ms (88% of VTCL) and an S2 pulse with an interval of 190 ms, successfully terminated the VT before charging. FVT = fast ventricular tachycardia; SVC = superior vena cava.

A 75-year-old man with a history of myocardial infarction and permanent atrial fibrillation received an ICD 20 years prior. With increased ventricular pacing, his New York Heart Association functional status deteriorated from class II to III, and the left ventricular ejection fraction (LVEF) decreased from 38% to 23%. Therefore, the patient underwent an upgrade to a cardiac resynchronization therapy defibrillator device (Cobalt XT HF CRTD; Medtronic). Given the patient’s recurrent VT episodes with presyncope, a 2-zone setting was selected as VT/VF therapy. Instead, repeated iATP therapy was programmed even in the VF zone (ie, ATP before charging ×2 and during charging ×1) (Figure 1A).

Six months after the cardiac resynchronization therapy defibrillator upgrade, the LVEF did not recover in this patient. One day, the patient visited the outpatient clinic with dyspnea and was diagnosed with worsening heart failure. On admission, a telemetry checkup revealed 1 VF episode, which was terminated by ATP therapies. However, 2 sequences of ATP therapies were required to terminate the VT in the VF zone (Figure 1B and 1C). Initially, the iATP algorithm administered the first sequence of the ATP for stable VT with a mean VTCL of 340 ms. This sequence consisted of 8 S1 pulses with a pacing cycle length (PCL) of 300 ms (88% of VTCL) and an S2 pulse with an interval of 280 ms. However, this ATP accelerated the VT, shortening the VTCL from 340 to 290 ms, categorizing it as tachycardia in the VF zone. The iATP algorithm then administered the first ATP sequence before charging. This sequence, comprising 10 S1 pulses with a PCL of 260 ms and an S2 pulse with an interval of 240 ms, altered but failed to terminate the VT. The second sequence, consisting of 12 S1 pulses with a PCL of 210 ms and an S2 pulse with an interval of 190 ms, successfully terminated the VT before charging. Although the time from the VF detection to termination was 9.4 seconds, the patient remained asymptomatic throughout the ATP therapy.

### Case 2

**Figure 2:**
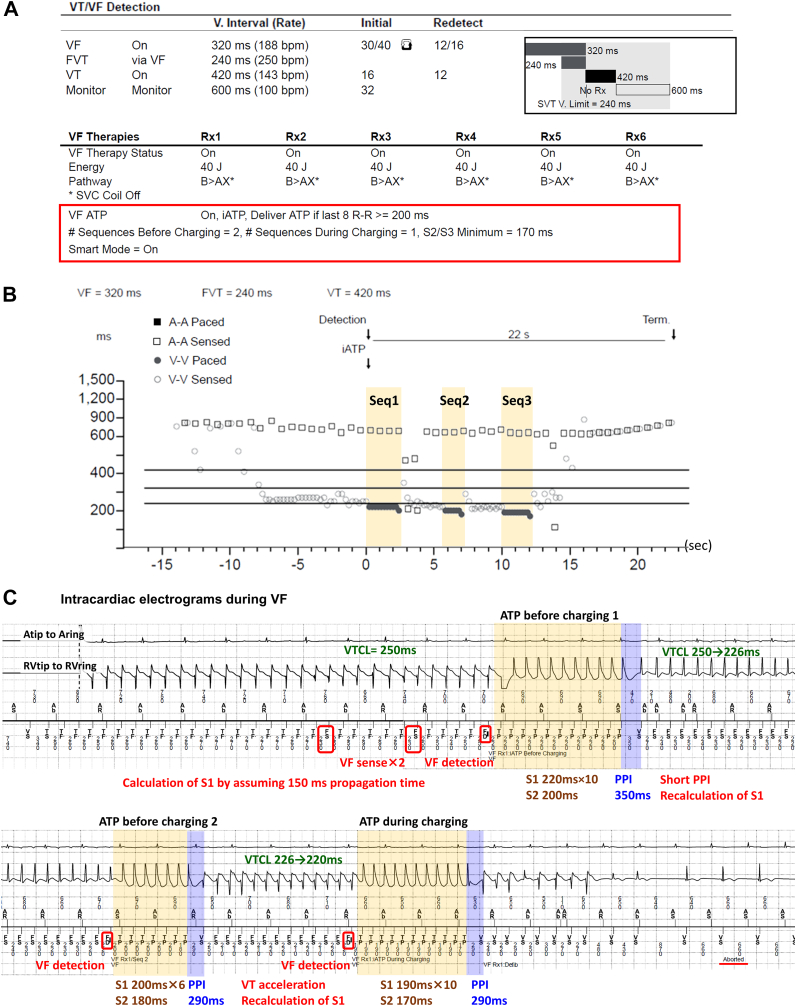
**A:** Parameter settings for tachycardia therapy in case 2. **B:** The Medtronic (Minneapolis, MN) programmer shows the successful administration of intrinsic antitachycardia pacing (ATP) for fast ventricular tachycardia (VT) within the ventricular fibrillation (VF) zone. ATP was delivered thrice (ie, ATP before charging ×2 and during charging ×1) (*yellow bands*), which eventually led to the tachycardia termination and prevented the occurrence of shock. **C:** Intracardiac electrograms during a VF episode. The *yellow* and *blue bands* represent the electrograms recorded during ATP and post-pacing intervals (PPIs). In this case, the initial ATP failed to terminate the VT. Based on the PPI, the intrinsic ATP (iATP) calculated the propagation time from the pacing site to the entrance of the VT circuit as (PPI – VTCL) / 2. The second ATP decreased the number of S1 pulses from 10 to 6, resulting in VT acceleration. Because the change in VTCL met the criteria (>10% change or >30-ms decrease in VTCL), the algorithm was reset. The third iATP sequence, comprising 10 S1 pulses with a pacing cycle length of 190 ms (88% of VTCL) and an S2 pulse with an interval of 170 ms, successfully terminated the VT during charging. FVT = fast ventricular tachycardia; SVC = superior vena cava.

A 51-year-old man with hypertrophic cardiomyopathy and a slightly reduced LVEF of 40% was admitted to our hospital due to a VT storm. Because the VT occurred repeatedly even after introducing amiodarone, we performed catheter ablation of the VT. Fortunately, the VT circuit was successfully identified on the epicardial surface and radiofrequency energy application at the isthmus terminated the VT. A secondary prevention ICD (Cobalt XT DR; Medtronic) was then implanted. Because the patient’s clinical VT was relatively slow and noninducible at the end of the ablation, repeated iATP therapy was programmed even in the VF zone (ie, ATP before charging ×2 and during charging ×1) (Figure 2A).

Approximately 10 months after the ICD implantation, the patient noticed a remote monitoring alarm and was admitted to the outpatient clinic. A telemetry checkup revealed 3 ventricular arrhythmia episodes (2 VTs and 1 VF). Fortunately, all 3 events were terminated by the ATP therapies, but 3 sequences of the ATP therapies were required to terminate the VF event (Figures 2B and 2C). Initially, a rapid VT with a mean VTCL of 250 ms was successfully identified as a fast VT. However, the shortest VTCL of 230 ms led to its classification as VF. The iATP algorithm administered the first sequence of the ATP before charging. It comprised 10 S1 pulses with a PCL of 220 ms (88% of VTCL) and an S2 pulse with an interval of 200 ms. This sequence altered but did not terminate the VT. The second sequence, comprising 6 S1 pulses with a PCL of 200 ms and an S2 pulse with an interval of 180 ms, also failed to terminate the VT. The third iATP sequence, consisting of 10 S1 pulses with a PCL of 190 ms and an S2 pulse with an interval of 170 ms, successfully terminated the VT during charging. Although the time from the detection to termination was 22 seconds, the patient remained asymptomatic throughout the ATP therapies.

## Discussion

The effectiveness of repeated ATP therapy in the VF zone remains uncertain due to concerns about VT acceleration, hemodynamic collapse, and delayed defibrillation. In the 2 cases presented, although the initial iATP accelerated the VT, subsequent ATP sequences successfully terminated fast VTs in the VF zone without inducing shock therapy. Although it is unclear whether the VT terminations were directly attributable to the iATP algorithm, our cases led us to reconsider the risks and benefits of programming repeated ATP therapies in the VF zones.

The current guidelines recommend ATP delivery before shock therapy in the VF zone if the VT rate is ≤230 beats/min.^3^^,^^4^ However, it remains unclear whether ATP should be administered when the VT rate exceeds 230 beats/min, and the optimal number of ATP sequences has yet to be determined. Retrospective analysis from the PainFree SST trial has demonstrated that programming additional ATP sequences can increase the success rate of VT termination.^1^^,^^2^ However, more than 3 ATP sequences for fast VTs (VTCL <320 ms) may provide minimal clinical benefit and could result in delayed defibrillation.

However, these findings are based on the conventional ATP algorithm. The novel iATP algorithm, specific to the Medtronic’s Cobalt XT device, automatically calculates the optimal number of S1 pulses and S2/S3 intervals based on the previous ATP therapies and post-pacing intervals.^5^^,^^6^ Initially, iATP set the S1 coupling interval to 88% of the VTCL. The number of S1 pulses is calculated on the basis of the pacing electrode-to-VT circuit travel time, assuming a default travel time of 150 ms for the initial sequences. If the initial ATP sequence fails to terminate VT, iATP makes 2 adjustments. First, it calculates the number of S1 pulses required to entrain the VT circuit using post-pacing interval and minimizes subsequent S1 pulses to the number needed for reset. If reset is not achieved, additional S1 pulses are added. Second, iATP estimates the refractory period during VT and progressively shortens the S2 interval until VT termination. Once loss of capture occurs, the S2 interval is restored to the shortest coupling interval that captures the myocardium, and an S3 pulse is added. Even if ATP therapy accelerates the VT (with >10% or >30-ms change in VTCL), the iATP algorithm resets the estimated conduction time (150 ms) and recalculates the number of S1 pulses based on the modified VT. This dynamic and adaptive programming may enhance the termination rate of fast VTs in the VF zone.^7^, ^8^, ^9^ Although iATP has shown greater effectiveness in slower VT compared with faster VT,^5^^,^^10^ it remains a valuable tool for managing fast VTs in the VF zone, aiming to terminate VT in the shortest possible time, even as the VT circuit evolves with the ATP.

Another important consideration is the number of repeated iATP sequences tolerated in the VF zone. Traditionally, ATP in the VF zone had been limited to a maximum of 2 sequences. Thus, this issue has not been thoroughly explored, even in the PainFree SST trial.^2^ However, with the introduction of the Cobalt XT device, up to 4 ATP sequences can now be programmed in the VF zone. The risks and benefits of this aggressive ATP therapy may vary based on patient characteristics, such as age, frailty, cardiac function, and comorbidities. As seen in case 1, relying solely on LVEF for risk stratification may be insufficient. In general, however, for patients with severely reduced LVEF (eg, LVEF <35%), repeated ATP therapy may increase the risk of delayed defibrillation and hemodynamic collapse. Conversely, for patients with moderately impaired LVEF (eg, LVEF >35%), as in case 2, repeated ATP therapy may prevent shock therapy and reduce patient discomfort. The type of ventricular arrhythmia is also a key factor influencing outcomes. While multiple burst ATP therapy has been found to be effective in terminating fast VT episodes,^11^ this is likely due to the fact that many arrhythmias classified in the VF zone are actually macro-reentrant VTs. Thus, for patients with channelopathies (eg, Brugada syndrome, long QT syndrome, and catecholaminergic polymorphic VT), who are more prone to occur VF rather than VT, repeated ATP therapy may not offer the same benefit. Adjusting device settings, such as increasing the R-R interval threshold for ATP delivery (eg, >240 ms in nominal setting), may improve safety. Large-scale multicenter studies are necessary to accurately assess the efficacy and safety of repeated ATP therapy in the VF zone.

## Conclusion

We encountered 2 cases in which the iATP algorithm was actively programmed in the VF zone, resulting in the successful termination of fast VTs through a dynamic optimization process. While repeated ATP therapy carries potential risks, such as VT acceleration and delayed defibrillation, it ultimately terminated the fast VT in our cases. Further research is needed to explore the efficacy and safety of programming repeated iATPs in the VF zone.

## Disclosures

The authors have no conflicts of interest to disclose.
